# HIF1α/HIF2α–Sox2/Klf4 promotes the malignant progression of glioblastoma via the EGFR–PI3K/AKT signalling pathway with positive feedback under hypoxia

**DOI:** 10.1038/s41419-021-03598-8

**Published:** 2021-03-24

**Authors:** Pan Wang, Lu Zhao, Sheng Gong, Shuanglong Xiong, Junwei Wang, Dewei Zou, Jinyu Pan, Yangmin Deng, Qian Yan, Nan Wu, Bin Liao

**Affiliations:** 1 Department of Neurosurgery, Chongqing General Hospital, University of Chinese Academy of Sciences, 401147 Chongqing, China; 2 Chongqing Medical University, 400016 Chongqing, China; 3 Department of Oncology, Chongqing University Cancer Hospital, 400030 Chongqing, China

**Keywords:** Cell growth, CNS cancer

## Abstract

Previous studies have suggested that hypoxic responses are regulated by hypoxia-inducible factors (HIFs), which in turn promote the malignant progression of glioblastoma (GBM) by inhibiting apoptosis and increasing proliferation; these events lead to a poor prognosis of GBM patients. However, there are still no HIF-targeted therapies for the treatment of GBM. We have conducted series of experiments and discovered that GBM cells exhibit features indicative of malignant progression and are present in a hypoxic environment. Knocking out HIF1α or HIF2α alone resulted in no significant change in cell proliferation and cell cycle progression in response to acute hypoxia, but cells showed inhibition of stemness expression and chemosensitization to temozolomide (TMZ) treatment. However, simultaneously knocking out HIF1α and HIF2α inhibited cell cycle arrest and promoted proliferation with decreased stemness, making GBM cells more sensitive to chemotherapy, which could improve patient prognosis. Thus, HIF1α and HIF2α regulate each other with negative feedback. In addition, HIF1α and HIF2α are upstream regulators of epidermal growth factor (EGF), which controls the malignant development of GBM through the EGFR–PI3K/AKT–mTOR–HIF1α signalling pathway. In brief, the HIF1α/HIF2α–EGF/EGFR–PI3K/AKT–mTOR–HIF1α signalling axis contributes to the growth of GBM through a positive feedback mechanism. Finally, HIF1α and HIF2α regulate Sox2 and Klf4, contributing to stemness expression and inducing cell cycle arrest, thus increasing malignancy in GBM. In summary, HIF1α and HIF2α regulate glioblastoma malignant progression through the EGFR–PI3K/AKT pathway via a positive feedback mechanism under the effects of Sox2 and Klf4, which provides a new tumour development model and strategy for glioblastoma treatment.

## Introduction

Glioblastoma (GBM) is located in a hypoxic microenvironment and undergoes malignant progression, which is mainly regulated by hypoxia-inducible factor-1α (HIF1α) and hypoxia-inducible factor 2α (HIF2α)^1^. In the regulation of GBM progression, HIF1α is universally expressed and plays a more significant role, while HIF2α shows restricted expression and presents a less important role^1^. Both HIF1α and HIF2α regulate GBM growth initially, but with tumour development, the regulation of HIF2α decreases, while HIF1α becomes increasingly important; eventually, GBM development is regulated by only HIF1α^1,2^, suggesting that targeting HIF1α alone can inhibit GBM growth. Unfortunately, this targeted therapy has not been successful thus far^3^. To explore the reason, we knocked out HIF1α and HIF2α individually or simultaneously and found that HIF1α and HIF2α exerted a mutual regulation on each other. In addition to HIF1α and HIF2α, epidermal growth factor (EGF) is highly expressed in GBM under hypoxic conditions^4,5^ and regulates GBM malignant progression through the EGFR–PI3K/AKT–mTOR signalling pathway^4,5^. A meaningful fact is that EGF has been shown to be an upstream gene-regulating HIF1α expression, and one of the downstream genes of the EGFR–PI3K/AKT–mTOR signalling pathway is HIF1α^6^, which indicates that EGF regulates HIF1α expression through the EGFR–PI3K/AKT–mTOR signalling pathway. Therefore, we studied and confirmed this regulatory mechanism. Another interesting result is that both HIF1α and HIF2α, as upstream genes, upregulated Sox2 and klf4 expression, indicating that HIF1α and HIF2α promote GBM malignant progression via the Sox2 and klf4 in hypoxia.

## Materials and methods

### Public data collection

Data were obtained from the Chinese Glioma Genome Atlas (CGGA, http://www.cgga.org.cn) and used to analyse the expression of HIF1α, HIF2α, EGF, EGFR, PI3K, PDK1, AKT, mTOR, Sox2 and Klf4 and the correlation among HIF1α, HIF2α and the above proteins through the dplyr, tibble and ggpolt2 packages in R.

### Patients and specimens

Three glioma tissues (GBM-1, WHO III; GBM-2, WHO IV; GBM-3, WHO IV) were obtained from surgical waste. The grade was pathologically verified after surgery, and detailed patient information is presented in Supplementary Table S6. All data relating to the patients were anonymized.

### Cell isolation and cell culture

GBM cells isolated from surgical tissue were used as cell lines. The detailed steps for the isolation of primary cells were in accordance with our previous studies^7^. Cells were cultured in DMEM/F12 (HyClone, USA) with 10% foetal bovine serum (FBS, Gibco, USA) to maintain growth at 37 °C.

### Immunofluorescence and western blotting

GBM cells were cultured in 21% O_2_ or 1% O_2_ for 72 h, and protein expression was detected via immunofluorescence and western blotting. The detailed steps for these assays were in accordance with our previous studies^7^, and information relating to the primary antibodies used is presented in Supplementary Tables S3 and S4.

### Real-time quantitative polymerase chain reaction (RT-qPCR)

GBM cells were cultured in 21% O_2_ or 1% O_2_ for 12 h to detect RNA expression through RT-qPCR. The primer sequences are presented in Supplementary Table S1, and the detailed steps for RT-qPCR were in accordance with our previous studies^7,8^.

### Flow cytometry (FCM) analysis

The cell cycle distribution and apoptotic cells were detected using FCM. For the cell cycle distribution, GBM cells were cultured in 21% O_2_ or 1% O_2_ for 72 h. For apoptosis detection, GMB cells were treated with temozolomide (TMZ, 400 μM) for 72 h after an initial 72 h of culture. The detailed steps for the cell cycle distribution and apoptosis assays were in accordance with our previous studies^7,8^.

### CCK-8 assay

Primary GBM cells were plated in 96-well plates (2000 cells/well) with DMEM/F12 + 10% FBS and cultivated in 21% O_2_ or 1% O_2_ for 72 h in the absence or presence of TMZ (400 μM). The detail steps for CCK-8 detection were in accordance with our previous studies^7^.

### EGF ELISA assays

Primary GBM cells were seeded at a density of 2 × 10^5^ cells per well in six-well plates with DMEM/F12 + 10% FBS and incubated in 21% O_2_ or 1% O_2_ for 72 h. Then, conditioned media were collected and stored at −80 °C. The EGF concentration in this media was detected with ELISA using a commercial kit (R&D Systems).

### Immunohistochemistry (IHC) detection

HIF1α and HIF2α expression in tumour tissues was detected by immunohistochemistry (IHC), and the detailed steps for IHC detection were in accordance with our previous studies^7^.

### Effect of EGFR, PI3K and mTOR inhibitors on GBM cells

Primary GBM cells were cultured in 1% O_2_ for 72 h, and inhibitors, including EGFR inhibitor (AG1478), PI3K inhibitor (Ly294002) and mTOR inhibitor (rapamycin), were added to the culture medium in a hypoxic environment for an additional 72 h. Cells were collected to detect HIF1α and HIF2α expression via western blotting.

### HIF1α, HIF2α, Sox2 and Klf4 knockout assays

The plasmid constructs for human HIF1α, HIF2α, Sox2 and Klf4 single guide RNAs (sgRNAs) (Supplementary Table S2) were designed based on the online CRISPR design program (http://crispr.mit.edu); these plasmid constructs were annealed and cloned into the lentiCRISPRv2 vector (#52961, Addgene, USA). Lentiviruses were transfected into 293T cells with the transducing vector, and then the packaging vectors psPAX2 (#12260, Addgene, USA) and pMD2.G (#12259, Addgene, USA) were used. After transfection for 48 h, the supernatant containing the virus particles was collected, filtered and transduced into GBM cells. Immunofluorescence and western blotting were used to confirm the knockout (KO) of HIF1α, HIF2α, Sox2 and Klf4.

### Microarray analysis

Empty vector cells, HIF1α-KO cells, HIF2α-KO cells and HIF1α/HIF2α-KO cells were cultured under hypoxic conditions for 24 h and were used for miRNA microarrays (GCBI, Shanghai, China). Raw microarray data have been saved in the NCBI Gene Expression Omnibus (GEO) database (www.ncbi.nlm.nih.gov/geo) under the accession number GSE142719. Signalling pathways with significant differences in this process were analysed according to the top 30 signalling pathways using KEGG with three comparing groups: empty vector cells and HIF1α-KO cells, empty vector cells and HIF2α-KO cells, empty vector cells and HIF1α/HIF2α-KO cells.

### Statistical analysis

Data are presented as the means ± standard deviation (SD), and SPSS 19.0 was used for statistical analyses. Significant differences between two groups were estimated using Student’s *t* test, and a one-way analysis of variance (ANOVA) was used for the comparison of at least three groups. Pearson’s correlation coefficient was used to analyse the correlations between genes. *P* < 0.05 was considered to be statistically significant.

## Results

### HIF1α/HIF2α is highly expressed in GBM under hypoxic conditions

**Fig. 1:**
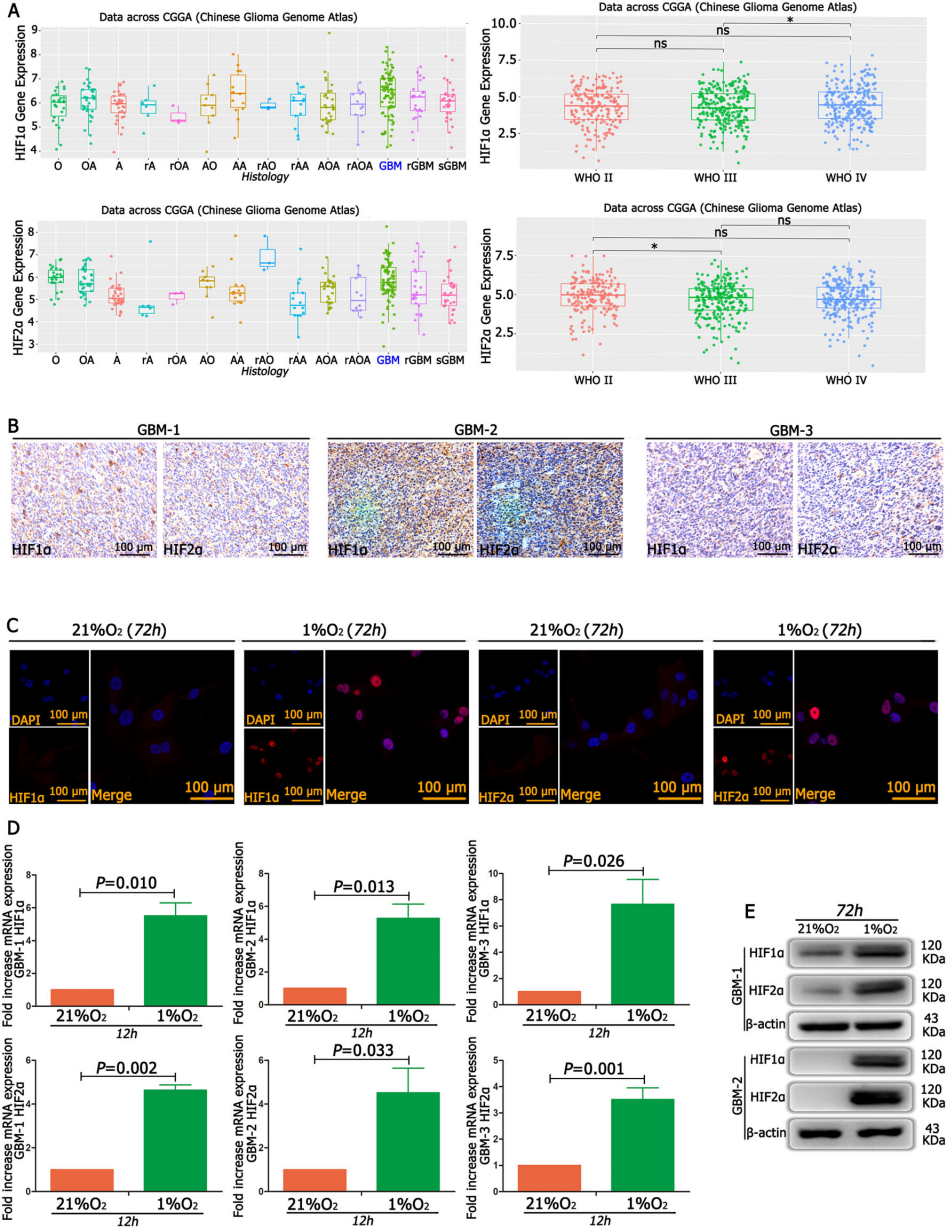
HIF1α and HIF2α were highly expressed in GBM under hypoxic conditions. **A** The CGGA database showed that both HIF1α and HIF2α were highly expressed in GBM. **B** Primary GBM tissues showed high levels of HIF1α and HIF2α expression using immunohistochemistry detection. **C** GBM cells exposed to 21% O_2_ and 1% O_2_ for 72 h showed that there was a much higher expression of HIF1α and HIF2α in cells cultured in 1% O_2_ than in cells cultured in 21% O_2_. **D**, **E** GBM cells cultured in 21% O_2_ and 1% O_2_ demonstrated that both HIF1α and HIF2α were highly expressed in 1% O_2_, while there was almost no HIF1α and HIF2α expression under 21% O_2_ conditions. *P* values were determined by the independent samples *t* test.

According to the CGGA database and IHC experiments, both HIF1α and HIF2α were highly expressed in GBM tissues (Fig. 1A, B and Supplementary Table S5). Then, GBM cells were cultured in 21% O_2_ and 1% O_2_ for 12 h, and the results from the RT-qPCR showed that HIF1α and HIF2α expression was much higher in the cells cultured in 1% O_2_ than in the control cells (Fig. 1D). We next cultured GBM cells in 21% O_2_ and 1% O_2_ for 72 h; in both western blotting and immunofluorescence results, both HIF1α and HIF2α were highly expressed under 1% O_2_ conditions, while there was almost no HIF1α and HIF2α expression under 21% O_2_ conditions (Fig. 1C, E).

### HIF1α and HIF2α regulated cell proliferation and apoptosis

**Fig. 2:**
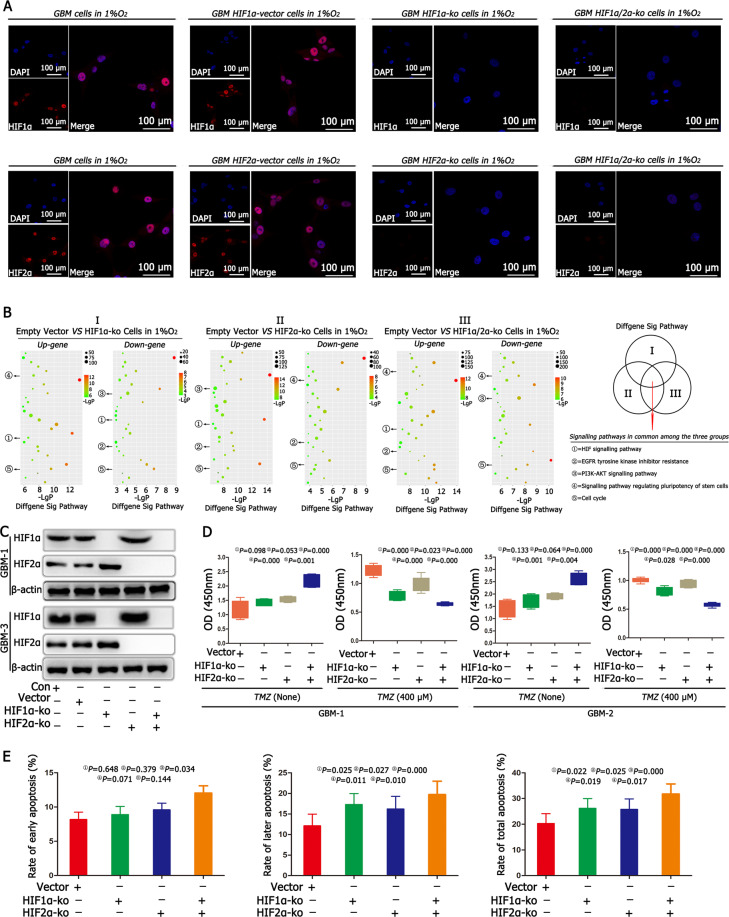
HIF1α and HIF2α regulated cell proliferation and apoptosis. **A** Immunofluorescence confirmed the successful knockout (KO) of HIF1α and HIF2α in HIF1α-KO, HIF2α-KO and HIF1α/HIF2α-KO cells. **B** We cultured empty vector cells, HIF1α-KO cells, HIF2α-KO cells, HIF1α/HIF2α-KO cells in 1% O_2_ for 24 h, KEGG pathway analysis revealed five common and significant signalling pathways, including the HIF signalling pathway, EGFR pathway, PI3K–AKT signalling pathway and signalling pathways regulating the pluripotency of stem cells and cell cycle using KEGG. **C** There were no differences in HIF1α and HIF2α between the control and empty vector groups after culturing both cells in 1% O_2_ for 72 h. However, the expression of HIF1α increased significantly after knocking out HIF2α; and HIF2α expression increased significantly after knocking out HIF1α. **D** After individually knocking out HIF1α or HIF2α, there were no differences in cell proliferation between the HIF1α-KO or HIF2α-KO cells and the empty vector cells. However, after simultaneously knocking out HIF1α and HIF2α, the cell proliferation rate increased significantly compared with the cell proliferation rates in other groups, including empty vector cells, HIF1α-KO cells or HIF2α-KO cells. Then, TMZ (400 μM) was added to the culture medium for another 72 h, and the cell proliferation became slower in HIF1α-KO or HIF2α-KO cells than in the empty vector cells; however, the slowest proliferation rate was found in the HIF1α/HIF2α-KO cells. **E** Cell apoptosis detection showed no difference in early apoptosis, but late and total apoptosis rates increased in HIF1α-KO or HIF2α-KO cells. However, after simultaneously knocking out HIF1α and HIF2α, there was a significant increase in early, late and total apoptosis rates compared with those in other groups. *P* values were determined by one-way ANOVA.

Immunofluorescence confirmed the successful KO of HIF1α and HIF2α (Fig. 2A and Supplementary Fig. S1). Empty vector cells, HIF1α-KO cells, HIF2α-KO cells, HIF1α/HIF2α-KO cells were cultured in 1% O_2_ for 24 h, and the commonly significant signalling pathways were analysed using KEGG. According to the results, we found that five signalling pathways were common and significant, including the HIF signalling pathway, EGFR tyrosine kinase inhibitor resistance pathway, PI3K–AKT signalling pathway, signalling pathways regulating the pluripotency of stem cells and cell cycle (Fig. 2B). We first focused on the regulatory mechanism of the HIF signalling pathway and found that there were no differences between the control and empty vector groups; however, the expression of HIF1α increased significantly after knocking out HIF2α, and HIF2α expression increased significantly after knocking out HIF1α (Fig. 2C). Then, we analysed cell proliferation without TMZ treatment. The results showed that after individually knocking out either HIF1α or HIF2α, there were no differences in cell proliferation between the HIF1α or HIF2α knockout group and the empty vector group. However, after simultaneously knocking out HIF1α and HIF2α, cell proliferation increased significantly when compared with cell proliferation in other three groups. Then, we added TMZ (400 μM) into the culture medium for another 72 h and found that cell proliferation became slower after individually knocking out HIF1α or HIF2α when compared with the cell proliferation in the empty vector group; however, the slowest proliferation rate was found in the HIF1α/HIF2α double KO group (Fig. 2D and Supplementary Fig. S2). In addition, we detected cell apoptosis, and the results showed that there were no differences in early apoptosis, but late and total apoptosis rates increased after individually knocking out either HIF1α or HIF2α when compared with the late and total apoptosis rates in the empty vector group. However, after simultaneously knocking out HIF1α and HIF2α, there was a significant increase in the early, late and total apoptosis rates when compared with these rates in other three groups (Fig. 2E, Supplementary Figs. S2 and S3A).

### HIF1α and HIF2α upregulated EGF in GBM under hypoxia

**Fig. 3:**
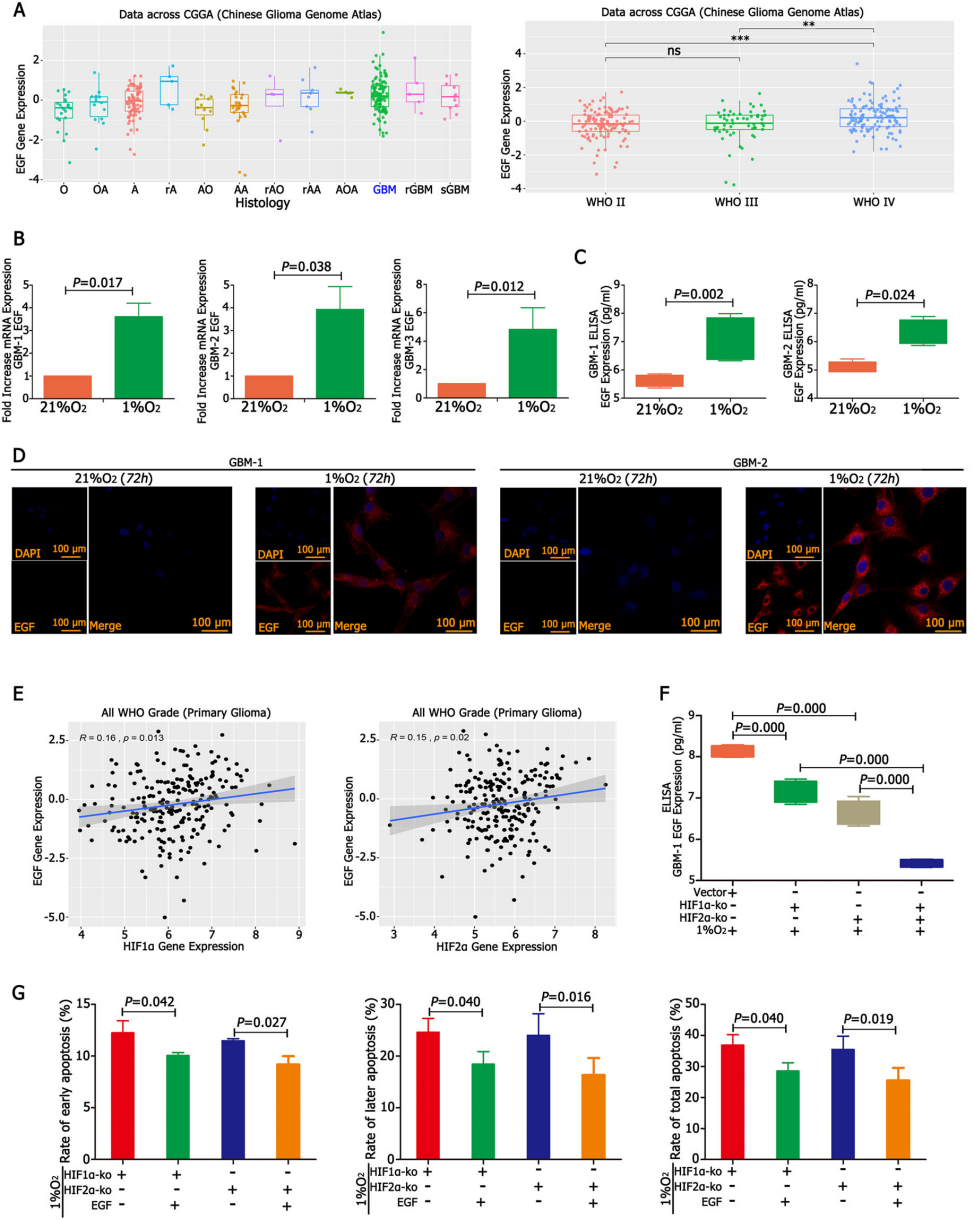
HIF1α and HIF2α upregulated EGF in hypoxia. **A** EGF was highly expressed in GBM, according to the CGGA database. **B** GBM cells were cultured in 21% O_2_ and 1% O_2_ for 12 h, and EGF expression was ~3.8–4.6-fold higher in cells cultured in 1% O_2_ than control cells cultured in 21% O_2_. **C** ELISA demonstrated that there were higher levels of EGF in the 1% O_2_ group than in the control group. **D** Immunofluorescence showed that there was no EGF expression under normoxia; however, EGF levels increased significantly under hypoxic conditions. **E** Both HIF1α and HIF2α had a positive relationship with EGF. **F** After knocking out HIF1α or HIF2α, EGF levels decreased significantly, and EGF expression was lowest after simultaneously knocking out HIF1α and HIF2α. **G** The addition of EGF had lower early, later and total apoptosis rates in HIF1α-KO or HIF2α-KO cells. *P* values were determined by the independent samples *t* test.

EGF was expressed in GBM according to the CGGA database, and WHO grade IV GBM had the highest EGF expression (Fig. 3A and Supplementary Table S5). Then, we cultured primary GBM cells in 21% O_2_ and 1% O_2_ for 12 h and RT-qPCR showed that EGF levels were ~3.8–4.6-fold higher in 1% O_2_ than in the control condition (Fig. 3B). ELISA demonstrated the same trends: there were much higher levels of EGF in cells in the 1% O_2_ group than in the control group (Fig. 3C). In addition, immunofluorescence demonstrated no EGF expression under normoxia; however, EGF was highly expressed under hypoxic conditions (Fig. 3D). Next, we analysed the relationship between HIF1α/HIF2α and EGF, which showed that both HIF1α and HIF2α had a positive relationship with EGF (Fig. 3E). Then, we detected EGF in HIF1α-KO and HIF2α-KO cells under hypoxia and found that mRNA levels decreased and after simultaneously knocking out HIF1α and HIF2α, EGF expression decreased significantly compared with the empty vector, HIF1α-KO and HIF2α-KO groups (Supplementary Fig. S4A). In addition, ELISA results found that after knocking out either HIF1α or HIF2α, EGF levels decreased significantly, and the lowest expression of EGF was observed after simultaneously knocking out HIF1α and HIF2α (Fig. 3F and Supplementary Fig. S4B). In addition, the apoptosis rate decreased after adding EGF to the culture medium of HIF1α-KO or HIF2α-KO cells (Fig. 3G and Supplementary Fig. S3B).

### HIF1α/HIF2α-EGF regulated GBM malignancy via the EGFR–PI3K/AKT pathway in hypoxia

**Fig. 4:**
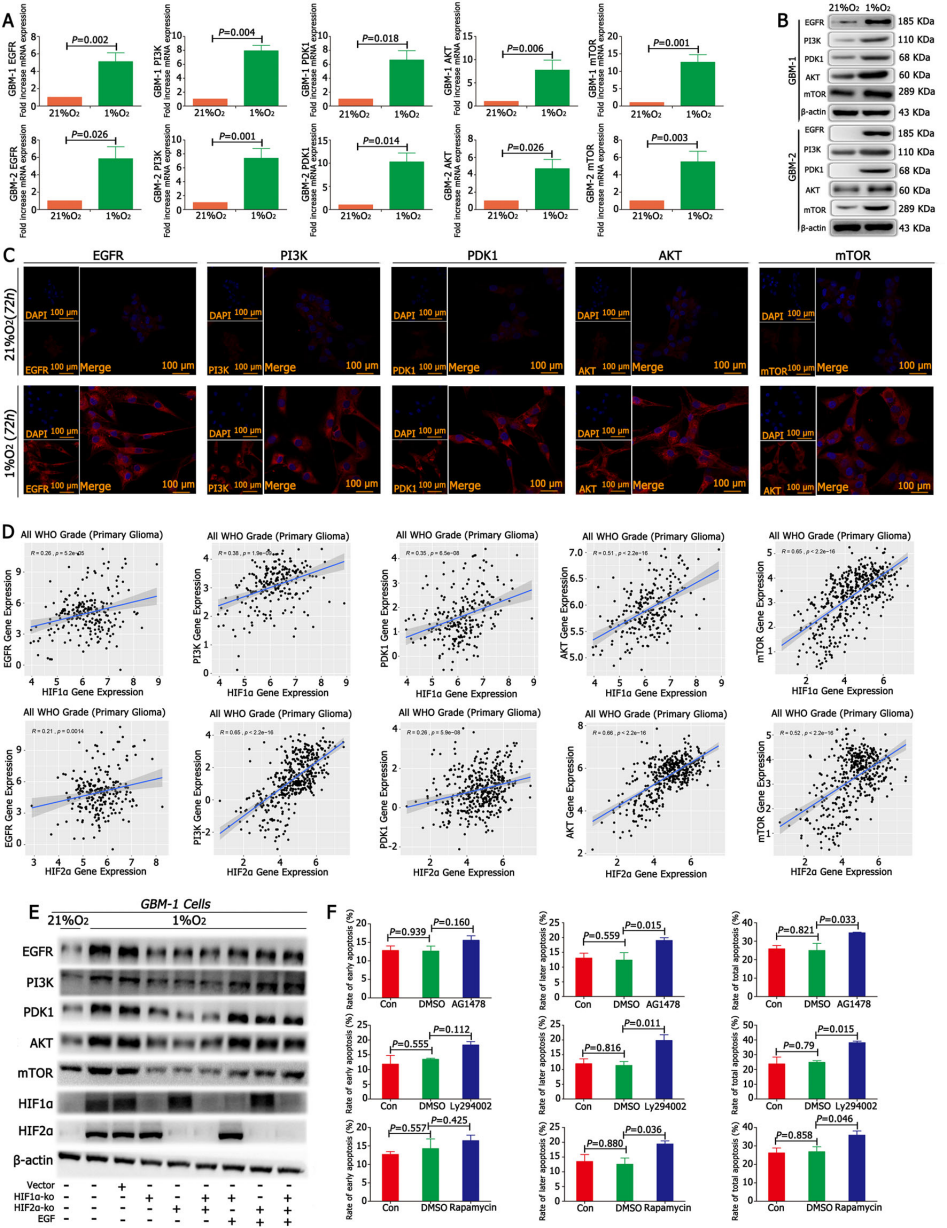
HIF1α/HIF2α-EGF regulated GBM malignancy via the EGFR–PI3K/AKT pathway in hypoxia. **A** There was a higher expression of EGFR, PI3K, PDK1, AKT and mTOR in cells cultured in 1% O_2_ than cells cultured in control conditions. **B**, **C** The cells cultured in 1% O_2_ for 72 h highly expressed EGFR, PI3K, PDK1, AKT and mTOR, but there was much less expression of EGFR, PI3K, PDK1, AKT and mTOR under normoxic conditions. **D** Both HIF1α and HIF2α had a positive correlation with EGFR, PI3K, PDK1, AKT and mTOR. **E** A significant decrease in the expression of EGFR, PI3K, PDK1, AKT and mTOR in HIF1α-KO or HIF2α-KO cells. The addition of EGF into the culture medium of HIF1α-KO or HIF2α-KO cells showed an immediate recovery of the expression of EGFR, PI3K, PDK1, AKT, mTOR and HIF1α. **F** Adding the EGFR inhibitor (AG1478), PI3K inhibitor (Ly294002) and mTOR inhibitor (rapamycin) into the culture medium of GBM cells in 1% O_2_ showed that there were no significant differences for early apoptosis; however, there were higher late and total apoptosis rates. *P* values were determined by the independent samples *t* test and one-way ANOVA.

According to the CGGA database, EGFR, PI3K, PDK1, AKT and mTOR were highly expressed in GBM (Supplementary Fig. S5 and Supplementary Table S5). Then, the cells were cultured in 21% O_2_ and 1% O_2_ for 12 h or 72 h to detect mRNA or protein expression. The results from RT-qPCR and western blotting showed that there was a higher expression of EGFR, PI3K, PDK1, AKT and mTOR in 1% O_2_ conditions than under control conditions (Fig. 4A, B). Immunofluorescence results showed that the cells in 1% O_2_ for 72 h highly expressed EGFR, PI3K, PDK1, AKT and mTOR, but there was no expression under normoxic conditions (Fig. 4C). Then, the relationship between HIF1α/HIF2α and the above proteins was analysed, and both HIF1α and HIF2α had a positive correlation with EGFR, PI3K, PDK1, AKT and mTOR (Fig. 4D). Furthermore, EGFR, PI3K, PDK1, AKT and mTOR were detected after knocking out either HIF1α or HIF2α. First, no differences in the expression of EGFR, PI3K, PDK1, AKT and mTOR were found between the control and empty vector groups in 1% O_2_; however, there was a significant decrease in the expression of EGFR, PI3K, PDK1, AKT and mTOR in HIF1α-KO and HIF2α-KO cells. The addition of EGF to the culture medium of HIF1α-KO or HIF2α-KO cells showed immediate recovery in the expression of EGFR, PI3K, PDK1, AKT, mTOR and HIF1α (Fig. 4E and Supplementary Fig. S6A). Previous studies have shown that the EGFR–PI3K–AKT–mTOR signalling pathway is an upstream regulator of HIF1α; this was verified in our study because we found that EGFR, PI3K and mTOR inhibitors (AG1478, Ly294002 and rapamycin, respectively) restrained HIF1α expression in GBM cells but showed no significant difference in HIF2α expression (Supplementary Fig. S6B). The apoptosis rate was detected after adding the above inhibitors into the medium of GBM cells in 1% O_2_, and the results demonstrated that there was no significant difference in early apoptosis rates; however, late and total apoptosis rates were higher in GBM cells in 1% O_2_ than in the control conditions (Fig. 4F and Supplementary Fig. S3C).

### Sox2, Klf4, CD133 and CD15 were expressed in GBM under hypoxia

**Fig. 5:**
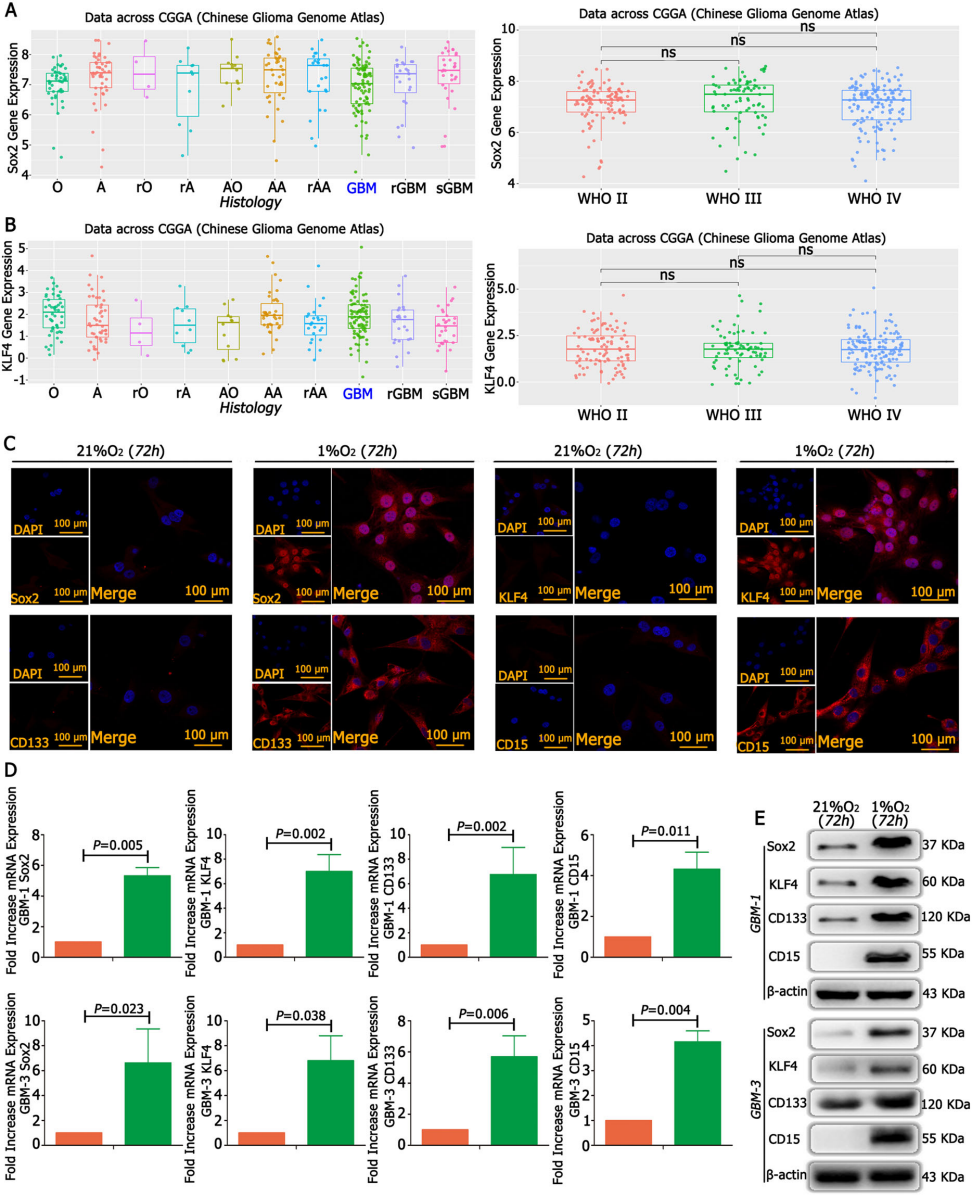
Sox2, Klf4, CD133 and CD15 were expressed in GBM under hypoxia. **A**, **B** Assessment of samples from the CGGA database showed that Sox2 and Klf4 were expressed in GBM. **C** GBM cells exposed to 21% O_2_ and 1% O_2_ for 72 h showed that Sox2, Klf4, CD133 and CD15 levels increased significantly in 1% O_2_ when compared with control cells in 21% O_2_. **D**, **E** There was a lower expression of Sox2, Klf4, CD133 and CD15 under normoxia, while these proteins increased significantly under hypoxic conditions. *P* values were determined by the independent samples *t* test.

According to the CGGA database, there was the expression of Sox2 and Klf4 in GBM (Fig. 5A, B and Supplementary Table S5), but there was no expression of Oct4, Nanog, Lin28A and Lin28B (Fig. S7). We cultured primary GBM cells in 21% O_2_ and 1% O_2_ for 12 h and detected mRNA expression of Sox2 and Klf4 through RT-qPCR, and the results showed that Sox2 and Klf4 increased significantly in 1% O_2_ compared with control conditions (Fig. 5D). Western blotting demonstrated the same trends; there were much higher levels of Sox2 and Klf4 in 1% O_2_ than under control conditions (Fig. 5E). We also used immunofluorescence to detect Sox2 and Klf4 and found no Sox2 and Klf4 expression under normoxia; however, Sox2 and Klf4 levels increased significantly under hypoxic conditions (Fig. 5C). In addition, we detected the expression of the stem cell markers CD133 and CD15 under hypoxic conditions, and the results demonstrated that the levels of CD133 and CD15 increased significantly under 1% O_2_ compared with normoxic conditions (Fig. 5C–E).

### HIF1α and HIF2α regulated GBM cell stemness expression through Sox2 and Klf4 under hypoxia

**Fig. 6:**
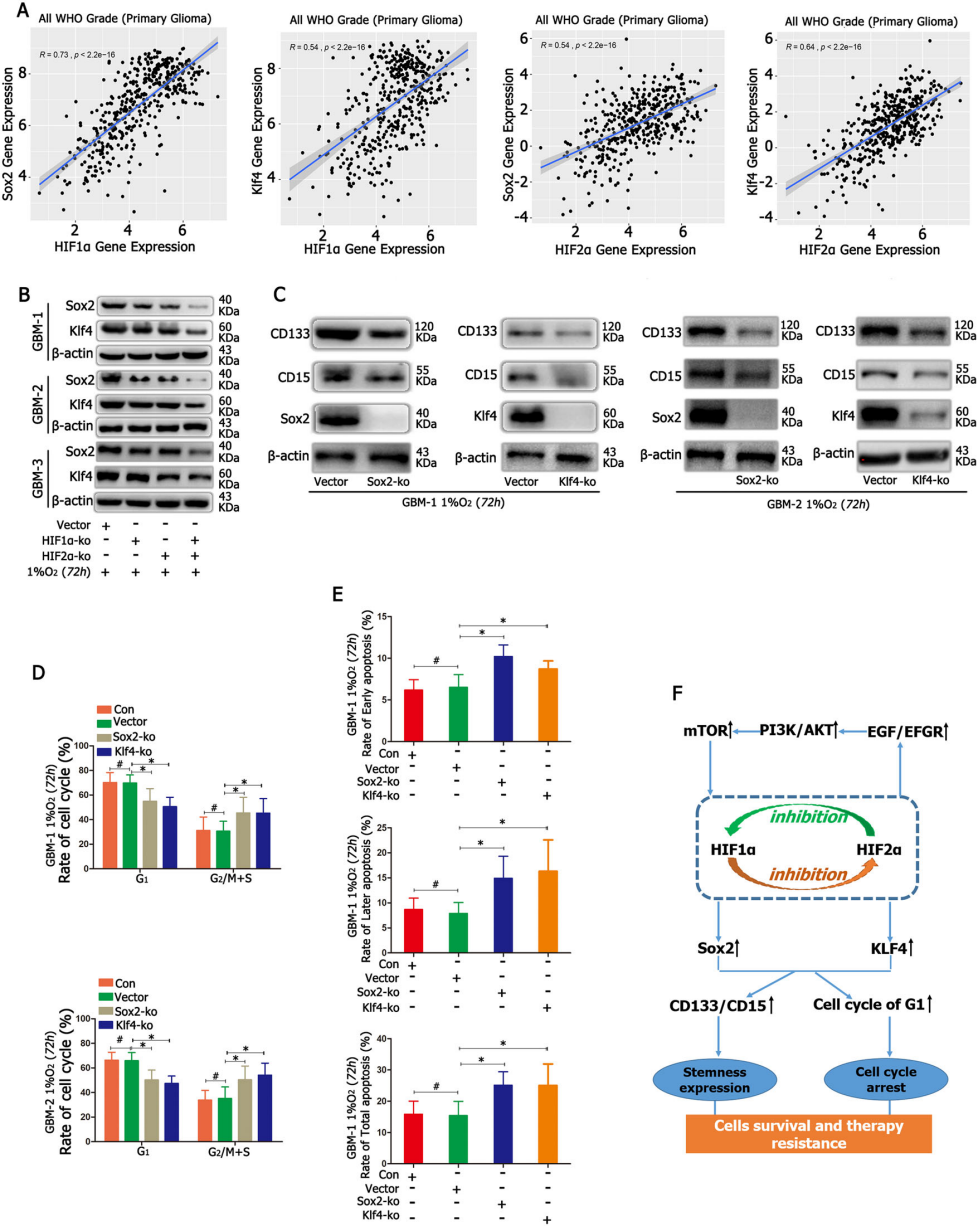
HIF1α and HIF2α regulated GBM cell stemness expression through Sox2 and Klf4 in hypoxia. **A** Both HIF1α and HIF2α had a positive relationship with Sox2 and Klf4. **B** There was a lower expression of Sox2 and Klf4 after knocking out HIF1α or HIF2α, and the levels were lowest after simultaneously knocking out HIF1α and HIF2α. **C** Compared with the control conditions, after knocking out Sox2 and Klf4, CD133 and CD15 expression decreased significantly. **D** The number of cells in the G_1_ phase decreased while the number of cells in the G_2_/M + S phase increased after knocking out Sox2 or Klf4. **E** After knocking out Sox2 or Klf4, the early, late and total apoptosis rates increased significantly compared with those in the control and empty vector cells. **F** HIF1α and HIF2α mutually regulate each other with negative feedback. HIF1α and HIF2α act as upstream gene regulators of EGF, and EGF regulates GBM malignancy through the EGFR–PI3K/AKT–mTOR–HIF1α signalling pathway. In addition, HIF1α and HIF2α upregulate Sox2 and Klf4 expression; high expression of Sox2 and Klf4 contributes to an increase in stemness expression and the transformation of cells in the G_2_/M + S phase to the G_1_ phase, thus leading to cell survival and therapy resistance. ^*^P < 0.05 and ^#^*P* > 0.05 were determined by one-way ANOVA.

First, the relationship between HIF1α/HIF2α and Sox2/Klf4 was analysed, and both HIF1α and HIF2α had a positive relationship with Sox2 and Klf4 (Fig. 6A). Then, we detected Sox2 and Klf4 expression for above groups in 1% O_2_, and the results showed in cells with the knockout of either HIF1α or HIF2α, there was a lower expression of Sox2 and Klf4 compared with that in the control cells; however, Sox2 and Klf4 levels were the lowest in cells with HIF1α and HIF2α knocked out (Fig. 6B). In addition, we were interested in whether Sox2 or Klf4 regulated CD133 and CD15; therefore, both Sox2 and Klf4 were knocked out individually, and these cells were cultured in 1% O_2_ for 72 h; the results showed that after knocking out Sox2 or Klf4, CD133 and CD15 levels decreased compared with the control conditions (Fig. 6C). Cell cycle arrest and chemotherapy resistance are other features of cancer stem cells, and we detected changes in the cell cycle after knocking out Sox2 or Klf4 under hypoxia. According to the graph, the number of cells in the G_1_ phase decreased, while the number of cells in the G_2_/M + S phase increased after knocking out Sox2 or Klf4 (Fig. 6D and Supplementary Fig. S8). Then, we added TMZ into the medium of Sox2-KO or Klf4-KO cells, and the results showed that after knocking out Sox2 or Klf4, the early, late and total apoptosis rates increased significantly compared with those of the control and empty vector cells (Fig. 6E and Supplementary Fig. S3D).

## Discussion

HIF1α and HIF2α are significant factors that contribute to the malignant progression of GBM^1,9^. However, one discrepancy from previous studies was that our experiment found that not only protein levels but also mRNA levels of HIF1α and HIF2α were increased under hypoxia. The reason we ascribe to this discrepancy is the different detection times used. We detected HIF1α and HIF2α mRNA expression under hypoxic conditions at 12 h, and our previous study^7,8^ showed that HIF1α and HIF2α mRNA levels increased in a time-dependent manner under hypoxia after <12 h. However, when cells were exposed to hypoxia for >24 h, the mRNA levels of HIF1α and HIF2α began to decrease while the protein levels increased^1,9^. Because of this, researchers think that if HIF1α or HIF2α is targeted successfully, GBM can be successfully treated. Nevertheless, to date, there have been no successful targeting therapies for HIF1α or HIF2α^3,10^. Therefore, we investigated cells with HIF1α or HIF2α knocked out individually or simultaneously. An interesting phenomenon was that knocking out HIF1α or HIF2α individually had no effect on GBM proliferation, but after knocking out HIF1α and HIF2α simultaneously, GBM grew; these results contradict the hypothesis that knocking out both HIF1α and HIF2α may inhibit tumour growth. However, after adding TMZ, the trends became different, showing a lower proliferation rate with an increase in cell apoptosis for HIF1α-KO or HIF2α-KO cells, and HIF1α/HIF2α-KO cells had the highest cell apoptosis, thus leading to the lowest proliferation.

Based on the core roles of HIF1α and HIF2α, we cultured empty vector cells, HIF1α-KO cells, HIF2α-KO cells and HIF1α/HIF2α-KO cells under hypoxia and analysed the main signalling pathways. Five pathways showed common differences among groups. The HIF signalling pathway was discussed above, and then discusses the relationship between the HIF1α/HIF2α and EGFR–PI3K/ATK signalling pathways. EGF promotes cell proliferation through the EGFR signalling pathway^11,12^. EGFR is also highly expressed in GBM^13^. Many pathways, such as the PI3K/AKT–mTOR pathway, can be activated under the influence of EGFR, leading to cell proliferation and stemness expression^14^. Moreover, HIF1α, as a downstream gene, can be regulated by the PI3K/AKT–mTOR signalling pathway^15,16^. That is, EGF induces HIF1α expression through the EGFR–PI3K/AKT–mTOR signalling pathway, thus regulating GBM growth. Significantly, in contrast to previous studies, this study found that in addition to HIF1α being a downstream gene of the EGF/EGFR–PI3K/AKT–mTOR signalling pathway, both HIF1α and HIF2α were upstream factors that upregulated the expression of EGF, EGFR, PI3K, AKT and mTOR. In brief, the HIF1α signalling pathway, EGFR tyrosine kinase inhibitor resistance pathway and PI3K–AKT signalling pathway regulate GBM progression under hypoxia through the HIF1α/HIF2α–EGF/EGFR–PI3K/AKT–mTOR–HIF1α network with positive feedback (Fig. 6F).

In addition, we focused on the effects of signalling pathways regulating the pluripotency of stem cells and the cell cycle on GBM malignant progression. Signalling pathways regulating the pluripotency of stem cells involve transcription factors include Sox2, Oct4, Nanog, Klf4, Lin28A and Lin28B, which are reprogrammed into many tissues such as human dermal fibroblasts^17^ and cardiomyocytes^18^, thus contributing to the formation of inducing pluripotent stem (iPS) cells. In addition to their roles in normal tissues, the abovementioned reprogramming factors in tumours such as colon cancer^19^, pancreatic cancer^20^, hepatocellular carcinoma^21^ and lung cancer^22^ induced the formation of cancer stem-like cells, and this process became remarkable under hypoxic conditions^23^, which is mainly regulated by HIF1α^24,25^. Therefore, we analysed the expression of the above proteins and found that only Sox2 and Klf4 were highly expressed in GBM. Regarding the relationship between HIF1α and Sox2/Klf4, previous studies have suggested that HIF1α upregulates Sox2 in GBM^26^ and Klf4 in mesenchymal stromal cells (MSCs)^27^ or colon cancer^24^; however, few reports have analysed the correlation between HIF1α and Klf4 in GBM. For the relationship between HIF2α and Sox2/Klf4, a few reports demonstrated that HIF2α upregulates Sox2 in embryonic stem cells (ESCs)^28^ and Klf4 in colon cancer^24^, but we do not know the correlation between HIF2α and Sox2 and Klf4 in GBM. To identify whether a correlation exists, we analysed the CGGA database and found that both HIF1α and HIF2α had a positive correlation with Sox2 and Klf4 in GBM. Then, HIF1α-KO and HIF2α-KO cells were cultured under hypoxia, and the results demonstrated that there was a much lower expression of Sox2 and Klf4. The results above suggested that both HIF1α and HIF2α, as upstream genes, upregulated Sox2 and Klf4 expression in GBM. Then, we knocked out Sox2 and Klf4 in GBM and demonstrated that the expression of the stem cell markers CD133 and CD15 decreased, which indicated that Sox2 and Klf4 induced stemness expression in GBM under hypoxia. Next, for the cell cycle, Otsubo et al.^29^ in 2008 found that Sox2 was downregulated in gastric cancers and inhibited cell growth through cell cycle arrest in the G_1_ phase. After overexpression of Sox2, the cell cycle progressed into the G_2_/M + S phase and promoted tumour growth. However, previous studies have contradictory results on GBM growth^30–32^; some studies showed that Sox2 contributed to GBM growth by promoting the cell cycle into the S phase, and knockdown of Sox2 attenuated S phase entry, thus inhibiting tumour growth^32,33^. However, in contrast, other studies showed that elevated Sox2 expression did not promote GBM cell proliferation, while knockdown of Sox2 induced GBM growth^30,31^. Klf4 was found to induce G_1_ phase arrest in Klf4-overexpressing cells, while S phase arrest was increased after repressing Klf4 expression in breast cancer^34^ and lung cancer^35^. However, few reports have demonstrated the influence of Klf4 on GBM growth and the cell cycle. Therefore, to clarify the effects of Sox2 and Klf4 on the cell cycle in hypoxia, we cultured cells for 72 h and found that Sox2-KO and Klf4-KO cells promoted cell cycle progression into the G_2_/M + S phase, thus stimulating GBM growth. Previous studies showed that the cells in G_2_/M + S phase^36^ or the cells decreased stemness properties^37^ are sensitised to chemotherapy; hence, Sox2-KO and Klf4-KO cells had higher apoptosis rates than did control cells in our study.

Briefly, we conclude that HIF1α and HIF2α regulate each other via negative feedback in GBM (Fig. 6F), which is the reason why targeting each one separately cannot ameliorate GBM patient prognosis. In addition, both HIF1α and HIF2α contribute to stemness expression and G_1_ phase arrest through Sox2 and Klf4, thus promoting GBM therapy resistance and a low proliferation rate (Fig. 6F). However, HIF1α- and HIF2α-KO cells induce the progression of the cell cycle into G_2_/M + S phase, thus promoting GBM growth while decreasing stemness, resulting in increased sensitivity of GBM cells to chemotherapy. Finally, HIF1α and HIF2α regulate the malignant progression of GBM through the EGFR–PI3K/AKT pathway with positive feedback (Fig. 6F), which provides a new tumour development model and a new strategy for treating GBM.

## Supplementary information

Sup_Figure_Legends

Table_S1

Table_S2

Table_S3

Table_S4

Table_S5

Table_S6

Sup Figure 1

Sup Figure 2

Sup Figure 3

Sup Figure 4

Sup Figure 5

Sup Figure 6

Sup Figure 7

Sup Figure 8
