# Supplementary material for: HIF1α/HIF2α–Sox2/Klf4 promotes the malignant progression of glioblastoma via the EGFR–PI3K/AKT signalling pathway with positive feedback under hypoxia
Source: Cell Death Dis. 2021 Mar 24;12(4):312. doi: 10.1038/s41419-021-03598-8 (PMC7990922; doi:10.1038/s41419-021-03598-8)
Supplement: Supplementary file 1 — Sup_Figure_Legends [file 41419_2021_3598_MOESM1_ESM.docx]

**Supplementary Figure S1** The results from immunofluorescence showed that HIF1α was highly expressed in HIF2α-ko cells under 1% O_2_ conditions, and HIF2α was highly expressed in HIF1α-ko cells under 1% O_2_ conditions.

**Supplementary Figure S2** *P* value from Figure 2.

**Supplementary Figure S3** The apoptosis results from the flow cytometry assays showing late apoptosis ([right](javascript:;) [upper](javascript:;) [quadrant](javascript:;)) and early apoptosis ([right](javascript:;) [lower](javascript:;) [quadrant](javascript:;)). The total apoptosis rate comprises the percentaes of cells in early and late apoptosis.

**Supplementary Figure S4 HIF1α and HIF2α upregulated EGF in GBM under hypoxia A** EGF mRNA levels decreased in HIF1α-KO or HIF2α-KO cells compared with the EGF expression in the empty vector group under 1% O_2_ conditions. However, after simultaneously knocking out HIF1α and HIF2α, EGF expression decreased significantly compared with the EGF expression in the three other groups, namely, the empty vector, HIF1α-KO and HIF2α-KO groups. **B** After knocking out HIF1α or HIF2α , EGF protein levels decreased significantly, and EGF expression was lowest after knocking out HIF1α and HIF2α.

**Supplementary Figure S5** The CGGA database showed that EGFR, PI3K, PDK1, AKT and mTOR were highly expressed in GBM.

**Supplementary Figure S6 HIF1α/HIF2α-EGF regulated the malignant progression of GBM through the EGFR-PI3K/AKT pathway under hypoxia A** Statistical graph of Figure 4E. **B** In GBM cells, the EGFR inhibitor (AG1478), PI3K inhibitor (Ly294002) and mTOR inhibitor (rapamycin) inhibited HIF1α expression but showed no significant difference in HIF2α expression.

**Supplementary Figure S7** There was no expression of Oct4, Nanog, Lin28A or Lin28B in GBM tissues.

**Supplementary Figure S8** Cell cycle distribution results of Figure 6D as determined with flow cytometry.

**Supplementary Table S1** The sequences of primers used for qRT-PCR experiments

**Supplementary Table S2** The sequences of sgRNAs used for knocking down HIF1α, HIF2α, Sox2 and Klf4

**Supplementary Table S3** Primary antibodies used in western blotting experiments

**Supplementary Table S4** Primary antibodies used in immunofluorescence experiments

**Supplementary Table S5** The abbreviations and full names for materials used in histological experiments in the article

**Supplementary Table S6** The detailed pathological information of the GBM patients assessed in this study
