# Supplementary material for: HIF1α/HIF2α–Sox2/Klf4 promotes the malignant progression of glioblastoma via the EGFR–PI3K/AKT signalling pathway with positive feedback under hypoxia
Source: Cell Death Dis. 2021 Mar 24;12(4):312. doi: 10.1038/s41419-021-03598-8 (PMC7990922; doi:10.1038/s41419-021-03598-8)
Supplement: Supplementary file 2 — Table_S1 [file 41419_2021_3598_MOESM2_ESM.docx]

Table S1 The sequences of primers used for qRT-PCR experiments

| EGF | Forward(5'-3') | GCCCCCCTGCCTCCTCCAAGTG |
| --- | --- | --- |
|  | Reverse(5'-3') | GGGGGTGGAGTAGAGTCAGGGCAA |
| EGFR | Forward(5'-3') | CCGCGCCCAGCAGAGACCCACAC |
|  | Reverse(5'-3') | AGTGGGCAGGGCTGTCGAATGTG |
| mTOR | Forward(5'-3') | CGCGCGAACCTCAGGGCAAGATG |
|  | Reverse(5'-3') | CCCTGGTTTCCTCATTCCGGCTCTT |
| PDK1 | Forward(5'-3') | TGGCCAAGGAGATCGAGGTCGCAGA |
|  | Reverse(5'-3') | CAACCTGCTCGGAACCCCTCTCTGT |
| PI3K | Forward(5'-3') | GCCGGTTCCGCCAGTGTTGTGAG |
|  | Reverse(5'-3') | TTTCCGAACTGTGTGGGCCATCC |
| AKT | Forward(5'-3') | GGCCCCCGAGGTGCTGGAGGACAA |
|  | Reverse(5'-3') | CGGGACCAAGCGTGCGCGGGAAG |
| HIF1A | Forward(5'-3') | ACTGCACAGGCCACATTCACG |
|  | Reverse(5'-3') | AATCAGCACCAAGCAGGTCATAGG |
| HIF2A | Forward(5'-3') | GCGACCATGAGGAGATTCGTGAG |
|  | Reverse(5'-3') | CAGGTGGCTGACTTGAGGTTGAC |
| CD15 | Forward(5'-3') | TGGGCAGGCTGGTCTTGAACT |
|  | Reverse(5'-3') | CACGGCGGCTCACACCTGTA |
| CD133 | Forward(5'-3') | GCCCCCAGGAAATTTGAGGAAC |
|  | Reverse(5'-3') | GCTTTGGTATAGAGTGCTCAGTGATTG |
| KLF4 | Forward(5'-3') | GGCTGCGGCAAAACCTACAC |
|  | Reverse(5'-3') | CGGGCGAATTTCCATCCAC |
| SOX2 | Forward(5'-3') | GGAGGGGTGCAAAAGAGGAGAG |
|  | Reverse(5'-3') | TCCCCCAAAAAGAAGTCCAGG |
| β-Actin | Forward(5'-3') | ACCCGCCGCCAGCTCACC |
|  | Reverse(5'-3') | GGGGGGCACGAAGGCTCATC |
