# Supplementary material for: HIF1α/HIF2α–Sox2/Klf4 promotes the malignant progression of glioblastoma via the EGFR–PI3K/AKT signalling pathway with positive feedback under hypoxia
Source: Cell Death Dis. 2021 Mar 24;12(4):312. doi: 10.1038/s41419-021-03598-8 (PMC7990922; doi:10.1038/s41419-021-03598-8)
Supplement: Supplementary file 3 — Table_S2 [file 41419_2021_3598_MOESM3_ESM.docx]

Table S2 The sequences of sgRNAs used for knocking down HIF1α, HIF2α, Sox2 and Klf4

| Target | Oligonucleotide sequence(5'-3') |
| --- | --- |
| HIF1A | GAACTCACATTATGTGGAAG |
| HIF2A | CTTGGAGGGTTTCATTGCCG |
| SOX2 | AAAGTTTCCACTCGGCGCCC |
| KLF4 | AGCGATACTCACGTTATTCGGGG |
