# Supplementary material for: HIF1α/HIF2α–Sox2/Klf4 promotes the malignant progression of glioblastoma via the EGFR–PI3K/AKT signalling pathway with positive feedback under hypoxia
Source: Cell Death Dis. 2021 Mar 24;12(4):312. doi: 10.1038/s41419-021-03598-8 (PMC7990922; doi:10.1038/s41419-021-03598-8)
Supplement: Supplementary file 4 — Table_S3 [file 41419_2021_3598_MOESM4_ESM.docx]

Table S3 Primary antibodies used in western blotting experiments

| Antigens | Manufacturer | Catalogue numbers | Application |
| --- | --- | --- | --- |
| HIF1A | abcam | ab179483 | 1:1000 |
| HIF2A | abcam | ab207607 | 1:1000 |
| CD15 | NOVUS | NB100-1831 | 2μg/ml |
| CD133 | NOVUS | NB120-16518 | 1:500 |
| EGFR | CST | 4267S | 1:1000 |
| PI3K | CST | 4249 | 1:1000 |
| PDK1 | CST | 5662S | 1:1000 |
| AKT | CST | 2920S | 1:1000 |
| mTOR | CST | 2587S | 1:1000 |
| SOX2 | CST | D9B8N | 1:1000 |
| KLF4 | R&D | AF3640 | 1:1000 |
| β-ctin | Beyotime | AF0003 | 1:1000 |
