# Supplementary material for: HIF1α/HIF2α–Sox2/Klf4 promotes the malignant progression of glioblastoma via the EGFR–PI3K/AKT signalling pathway with positive feedback under hypoxia
Source: Cell Death Dis. 2021 Mar 24;12(4):312. doi: 10.1038/s41419-021-03598-8 (PMC7990922; doi:10.1038/s41419-021-03598-8)
Supplement: Supplementary file 6 — Table_S5 [file 41419_2021_3598_MOESM6_ESM.docx]

Table S5 The abbreviations and full names for materials used in histological experiments in the article

| abbreviation | Full Name |
| --- | --- |
| O | oligodendrocytoma |
| OA | oligoastrocytoma |
| A | [astrocytoma](javascript:;) |
| rA | Recurrent [astrocytoma](javascript:;) |
| rOA | Recurrent oligoastrocytoma |
| AO | Anaplastic oligodendrocytoma |
| AA | Anaplastic astrocytoma |
| rAO | Recurrent anaplastic oligodendrocytoma |
| rAA | Recurrent anaplastic astrocytoma |
| AOA | Anaplastic oligoastrocytoma |
| rAOA | Recurrent anaplastic oligoastrocytoma |
| GBM | Glioblastoma multiforme |
| rGBM | Recurrent glioblastoma multiforme |
| sGBM | Secondary glioblastoma multiforme |
