# Supplementary material for: HIF1α/HIF2α–Sox2/Klf4 promotes the malignant progression of glioblastoma via the EGFR–PI3K/AKT signalling pathway with positive feedback under hypoxia
Source: Cell Death Dis. 2021 Mar 24;12(4):312. doi: 10.1038/s41419-021-03598-8 (PMC7990922; doi:10.1038/s41419-021-03598-8)
Supplement: Supplementary file 7 — Table_S6 [file 41419_2021_3598_MOESM7_ESM.docx]

|  | | GBM-1 | GBM-2 | GBM-3 |
| --- | --- | --- | --- | --- |
| Sex | | male | male | male |
| Age | | 57 years old | 46 years old | 52 years old |
| Primary or recurrent tumor | | Primary tumor | Primary tumor | Primary tumor |
| Pathology | GFAP | + | + | + |
|  | Olig-2 | + | + | + |
|  | Ki67 | 10% | 40% | 30% |
|  | P53 | + | \ | + |
|  | MGMT Methylation | - | + | - |
|  | IDH1-132 | Wild type | Wild type | Wild type |
|  | IDH1-127 | Wild type | Wild type | Wild type |
|  | IDH2-172 | Wild type | Wild type | Wild type |
|  | 1p/19q Codeletion | - | - | - |
| Integrated  diagnosis | | WHO III,  IDH-1/2 Wild type | WHO IV,  IDH-1/2 Wild type | WHO IV,  IDH-1/2 Wild type |

Table S6 The detailed pathological information of the GBM patients assessed in this study

Content

Tissue
