# Supplementary figures and images for: HIF1α/HIF2α–Sox2/Klf4 promotes the malignant progression of glioblastoma via the EGFR–PI3K/AKT signalling pathway with positive feedback under hypoxia
Source: Cell Death Dis. 2021 Mar 24;12(4):312. doi: 10.1038/s41419-021-03598-8 (PMC7990922; doi:10.1038/s41419-021-03598-8)

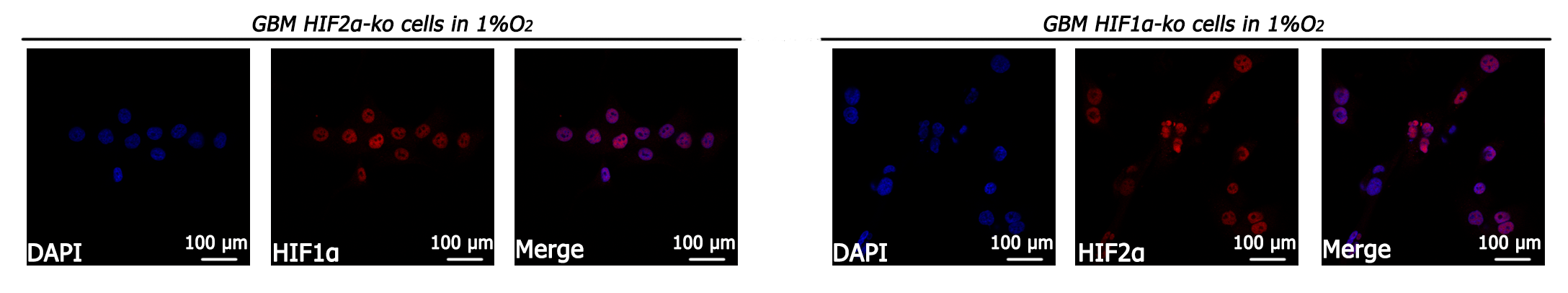

Supplement: Supplementary file 8 — Sup Figure 1 [file 41419_2021_3598_MOESM8_ESM.tif]

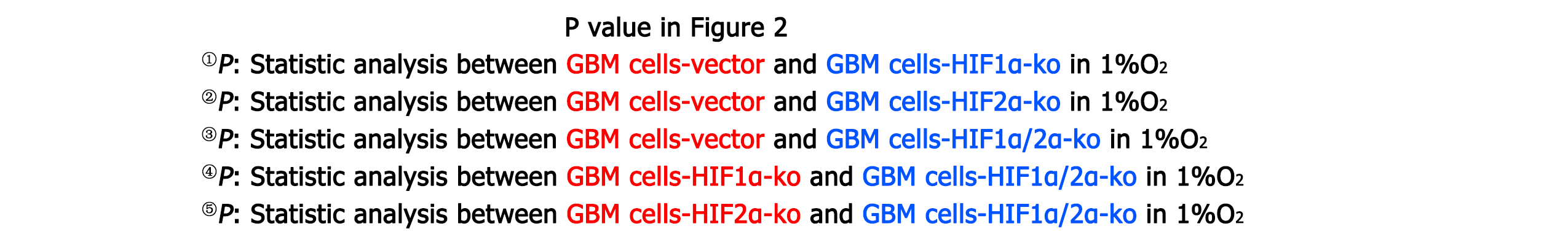

Supplement: Supplementary file 9 — Sup Figure 2 [file 41419_2021_3598_MOESM9_ESM.tif]

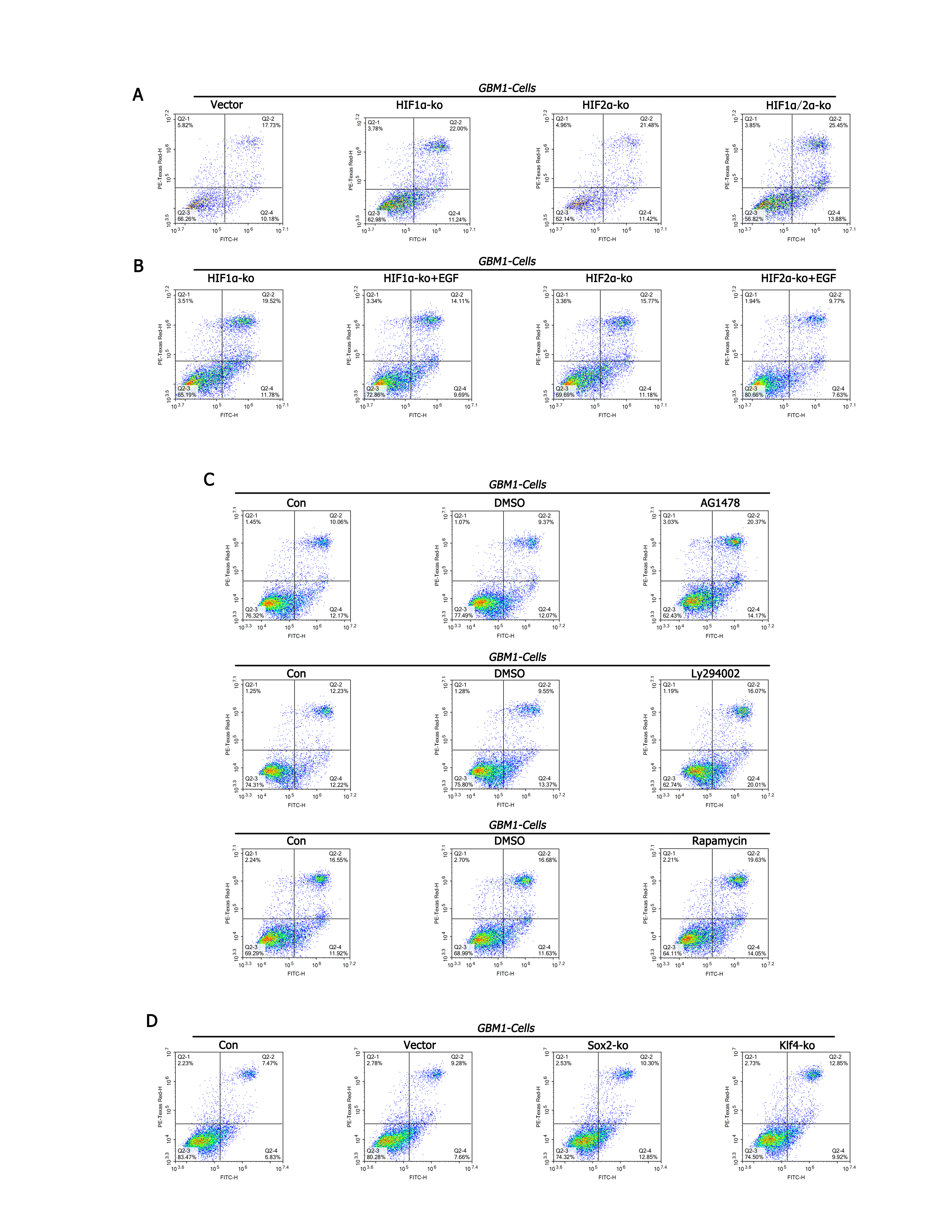

Supplement: Supplementary file 10 — Sup Figure 3 [file 41419_2021_3598_MOESM10_ESM.tif]

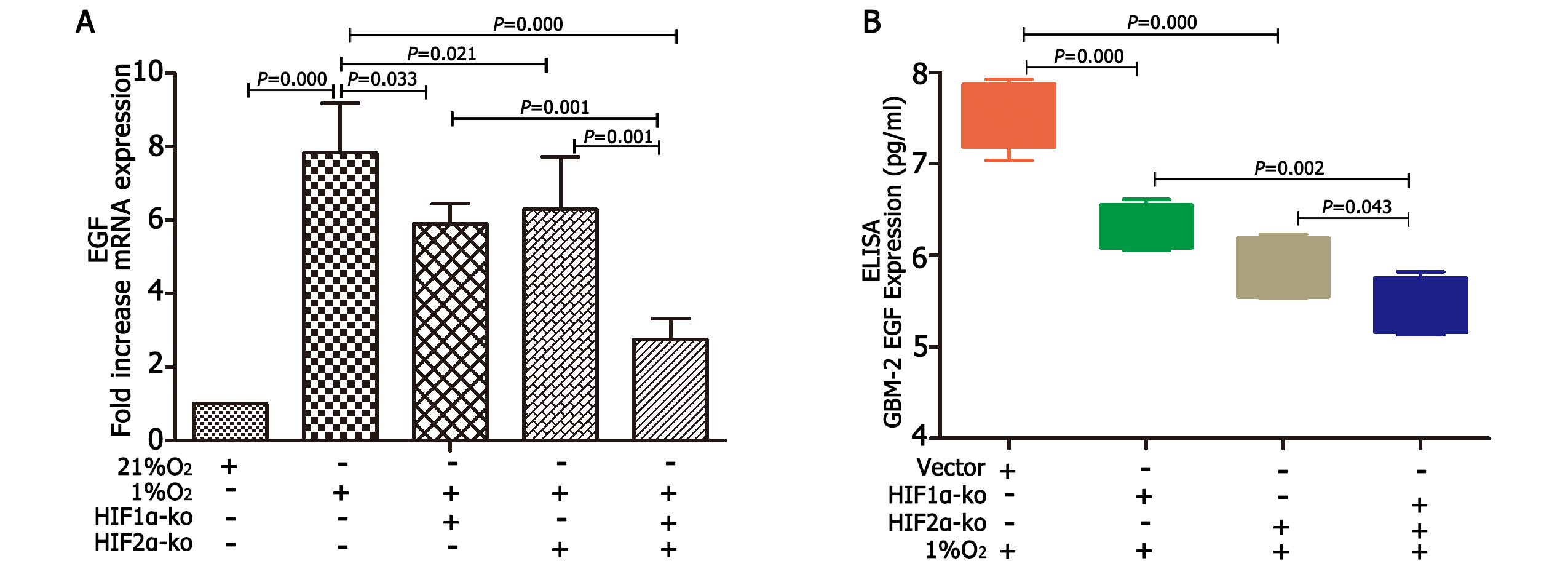

Supplement: Supplementary file 11 — Sup Figure 4 [file 41419_2021_3598_MOESM11_ESM.tif]

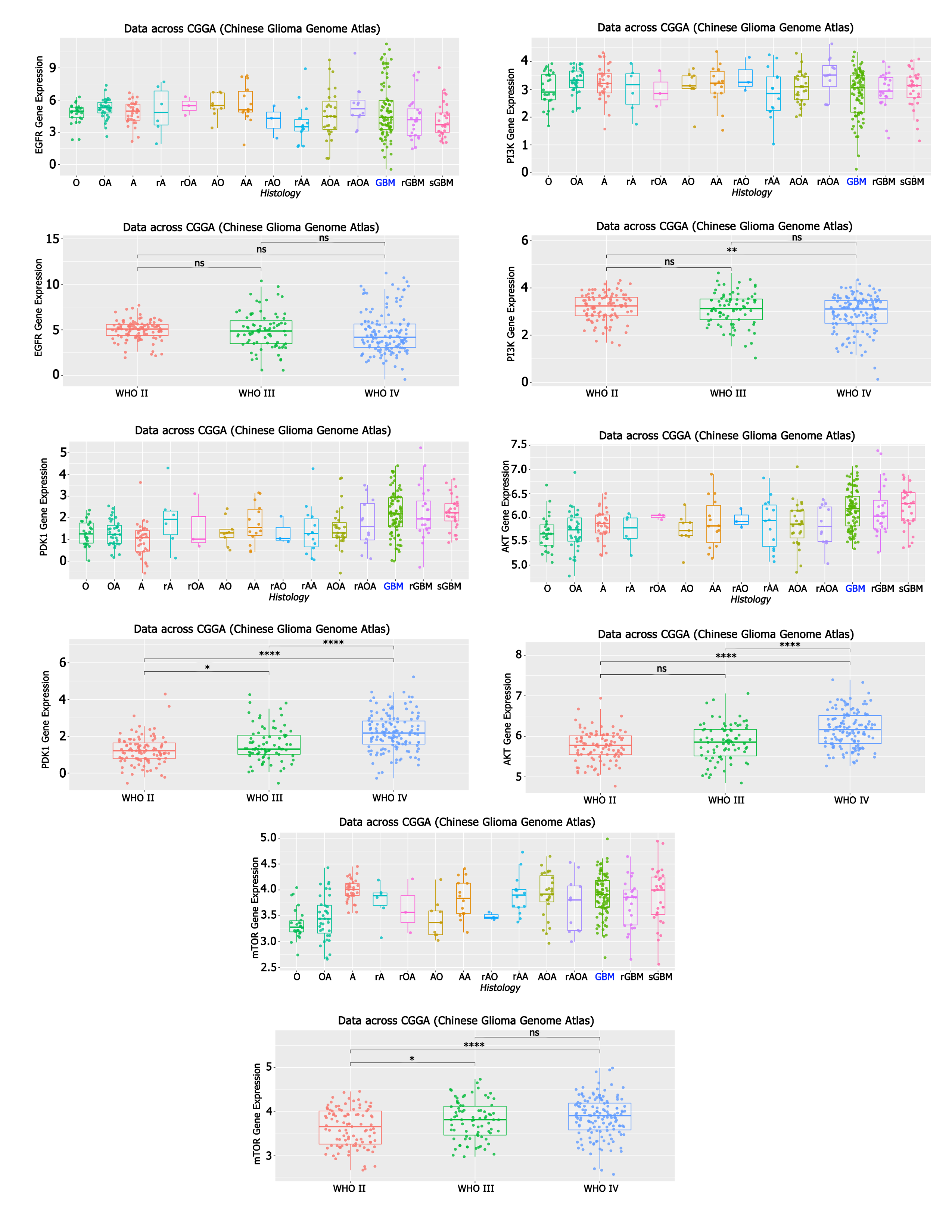

Supplement: Supplementary file 12 — Sup Figure 5 [file 41419_2021_3598_MOESM12_ESM.tif]

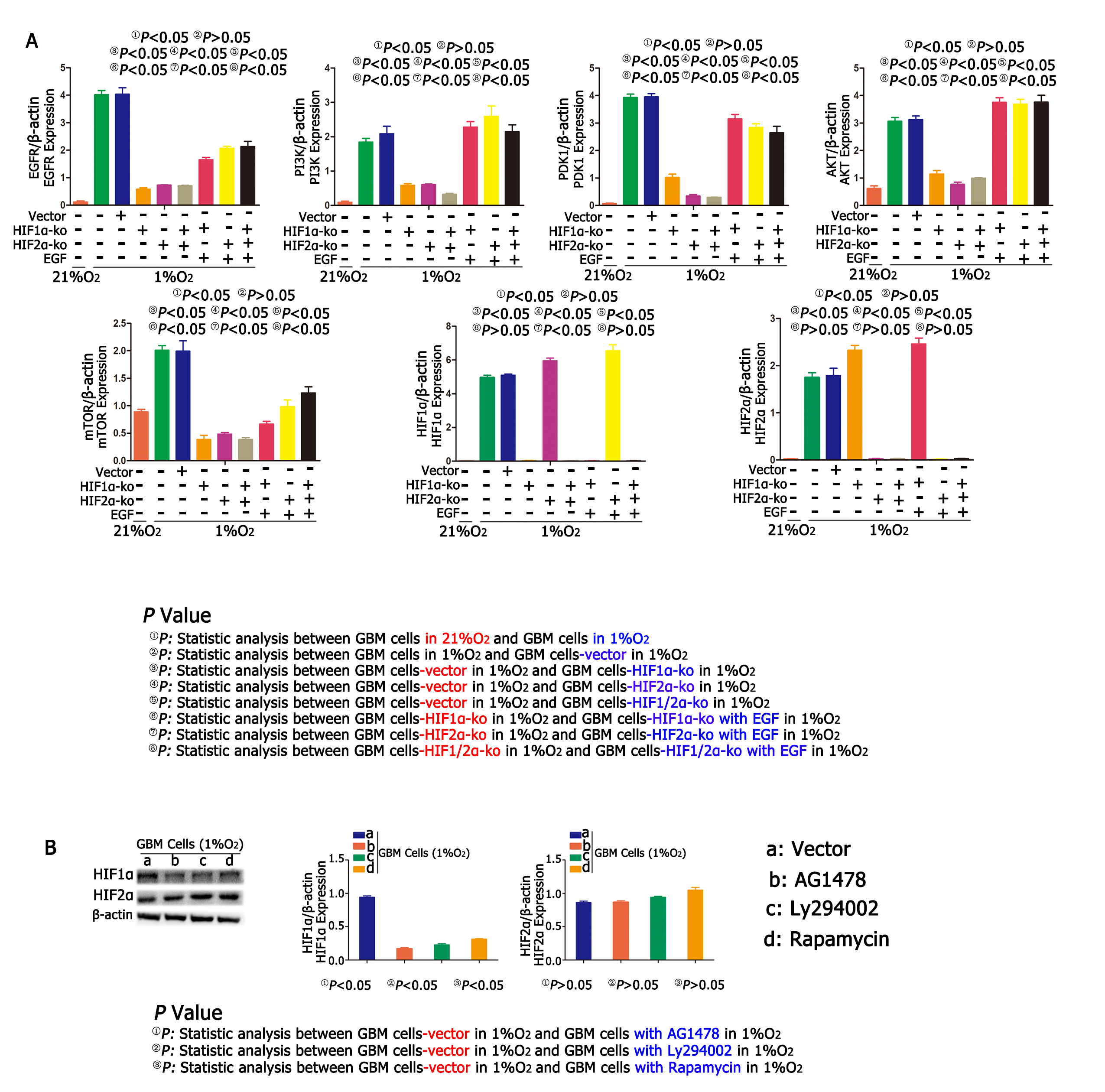

Supplement: Supplementary file 13 — Sup Figure 6 [file 41419_2021_3598_MOESM13_ESM.tif]

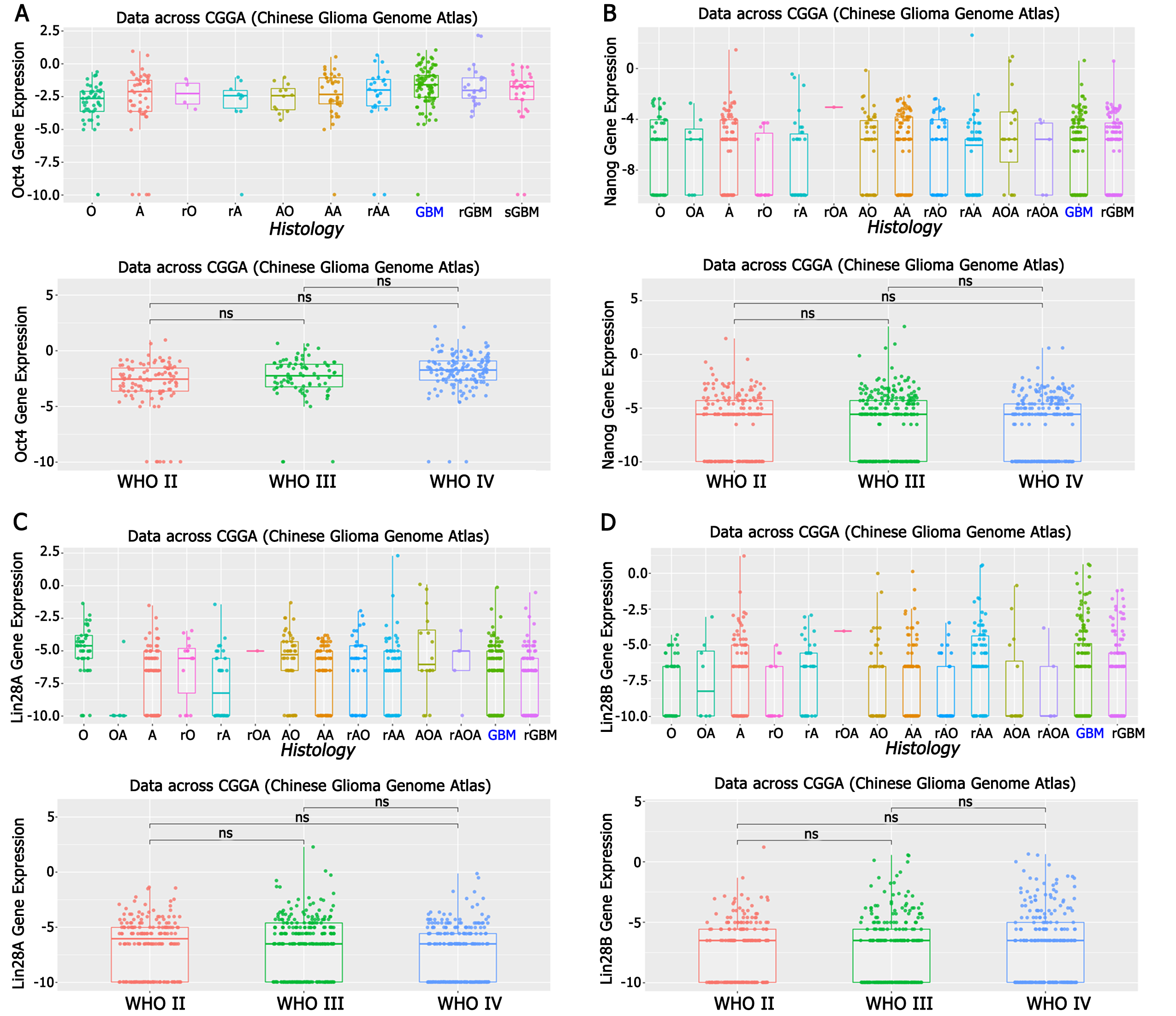

Supplement: Supplementary file 14 — Sup Figure 7 [file 41419_2021_3598_MOESM14_ESM.tif]

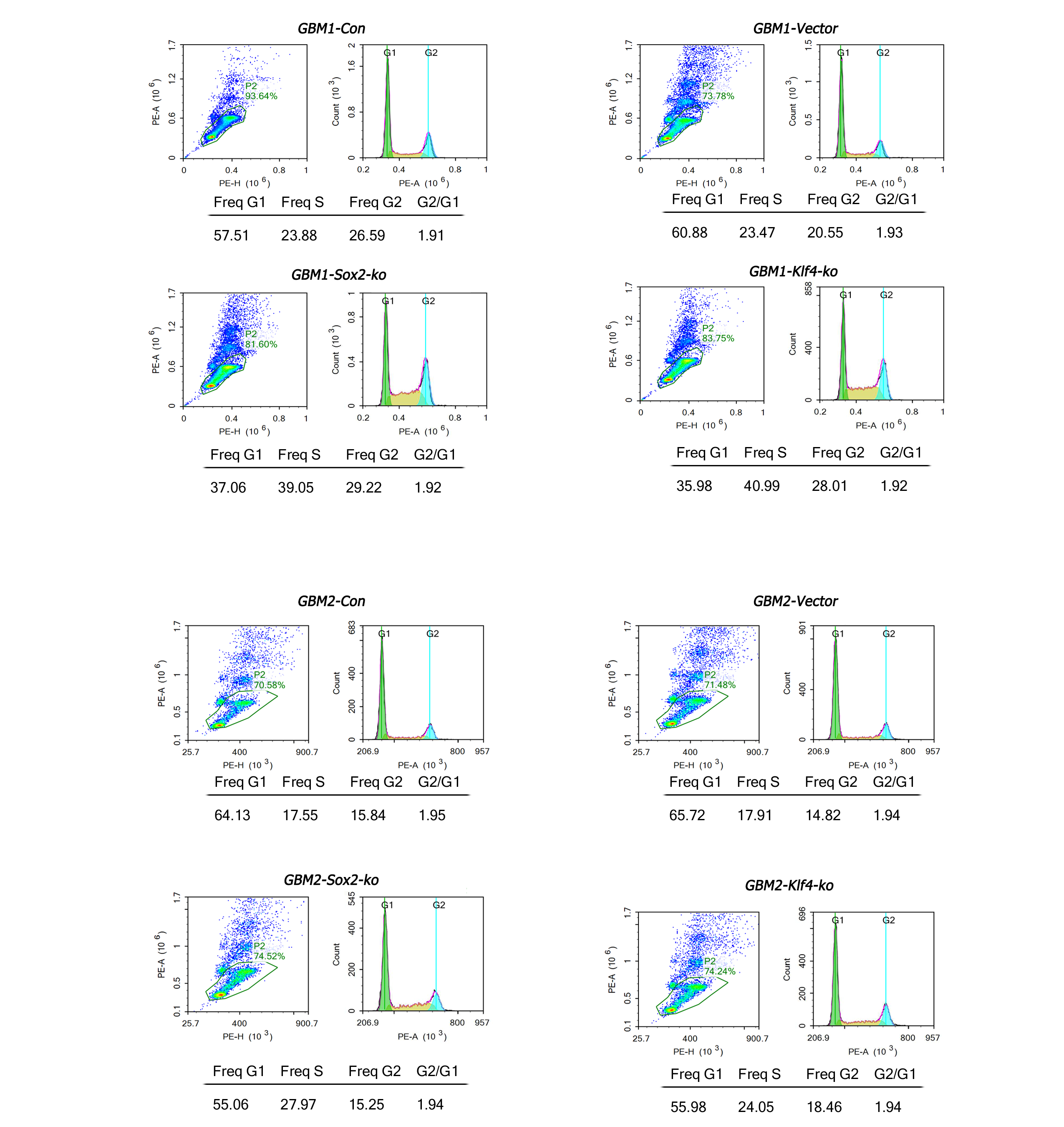

Supplement: Supplementary file 15 — Sup Figure 8 [file 41419_2021_3598_MOESM15_ESM.tif]
